# Which Hyperglycemic Model of Zebrafish (*Danio rerio*) Suites My Type 2 Diabetes Mellitus Research? A Scoring System for Available Methods

**DOI:** 10.3389/fcell.2021.652061

**Published:** 2021-03-15

**Authors:** Aria Salehpour, Mohammad Rezaei, Arezoo Khoradmehr, Yaser Tahamtani, Amin Tamadon

**Affiliations:** ^1^The Persian Gulf Marine Biotechnology Research Center, The Persian Gulf Biomedical Sciences Research Institute, Bushehr University of Medical Sciences, Bandar Bushehr, Iran; ^2^Department of Diabetes, Obesity and Metabolism, Cell Science Research Center, Royan Institute for Stem Cell Biology and Technology, Academic Center for Education, Culture and Research, Tehran, Iran; ^3^Department of Stem Cells and Developmental Biology, Cell Science Research Center, Royan Institute for Stem Cell Biology and Technology, Academic Center for Education, Culture and Research, Tehran, Iran; ^4^Reproductive Epidemiology Research Center, Royan Institute for Reproductive Biomedicine, Academic Center for Education, Culture and Research, Tehran, Iran; ^5^Center of Marine Experimental and Comparative Medicine, The Persian Gulf Marine Biotechnology Research Center, The Persian Gulf Biomedical Sciences Research Institute, Bushehr University of Medical Sciences, Bandar Bushehr, Iran

**Keywords:** zebrafish, *Danio rerio* (zebrafish), diabetes mellitus, T2DM, hyperglycemia, animal modeling

## Abstract

Despite extensive studies on type 2 diabetes mellitus (T2DM), there is no definitive cure, drug, or prevention. Therefore, for developing new therapeutics, proper study models of T2DM is necessary to conduct further preclinical researches. Diabetes has been induced in animals using chemical, genetic, hormonal, antibody, viral, and surgical methods or a combination of them. Beside different approaches of diabetes induction, different animal species have been suggested. Although more than 85% of articles have proposed rat (genus *Rattus*) as the proper model for diabetes induction, zebrafish (*Danio rerio*) models of diabetes are being used more frequently in diabetes related studies. In this systematic review, we compare different aspects of available methods of inducing hyperglycemia referred as T2DM in zebrafish by utilizing a scoring system. Evaluating 26 approved models of T2DM in zebrafish, this scoring system may help researchers to compare different T2DM zebrafish models and select the best one regarding their own research theme. Eventually, glyoxalase1 (glo1^−/−^) knockout model of hyperglycemia achieved the highest score. In addition to assessment of hyperglycemic induction methods in zebrafish, eight most commonly proposed diabetic induction approval methods are suggested to help researchers confirm their subsequent proposed models.

## Introduction

Zebrafish, nowadays known as a preferable animal model, was first introduced as an laboratory animal by Streisinger et al. (1981). They described zebrafish as a favorable model for developmental studies due to its transparency of embryo and remarkable fecundity (Streisinger et al., 1981; Grunwald and Streisinger, 1992). After a while, large-scale genetic studies demonstrated that zebrafish conserves ortholog genes which get disrupted in the state of illness similar to humans (Haffter et al., 1996; Barbazuk et al., 2000). In a short time, zebrafish converted to a frequent model of disease and drug discovery simultaneously with progression of genetic methods (Driever et al., 1996; Serbedzija et al., 1999; Dooley and Zon, 2000; Nasevicius and Ekker, 2000). It drew a lot of attention to be a proper model for metabolic diseases (Wang et al., 1998).

A variety of key features like resembling mammalian like pancreas specifications (Zon and Peterson, 2005), having main organ systems, such as a beating heart, mammalian like glucose regulation (Jurczyk et al., 2011), and similarity of nearly 70% of genes to human (Howe et al., 2013) make zebrafish superior in many scenarios. In addition, whole organ transcriptomic (Junker et al., 2014) and proteomic analyses (Nolte et al., 2015) are possible due to small size and in large scales due to laying capabilities of zebrafish. Zebrafish digestion system, adipose tissues, and skeletal muscle are physically humanlike (Schlegel and Stainier, 2007). Furthermore, blood circulation in vessels and neuronal and hormonal systems develop and become functional at early stages of development and make zebrafish favorable for early metabolic studies (Kimmel et al., 1995; Lakstygal et al., 2019; Pourghadamyari et al., 2019).

Scientists found it interesting to study type 2 diabetes mellitus (T2DM) in zebrafish as an animal model. T2DM is a very common metabolic disorder that can progress to other disorders, such as neurodegeneration (Sheikhpour and Khoradmehr, 2014; Farhadi et al., 2019). Scientists preferred utilizing rodents for preclinical studies of diabetes (Hajizadeh et al., 2014; Hajizadeh-Saffar et al., 2015; Engel et al., 2019). Actually, more than 85% of articles have used rat as their preferred model (Tripathi and Verma, 2014). So far, type 1 diabetes mellitus (T1DM), T2DM, and maturity-onset diabetes of the young (MODY) have been induced by a variety of methods in zebrafish (Zang et al., 2018).

Among the proposed methods, genetic methods may be more precise to target specific genes and produce abnormalities that are more accurate and more specific in results in comparison to other means. However, non-genetic ways of diabetes induction may be more available, cheaper, or easier to induce. In this systematic review, we aim to evaluate and compare all the available approaches for induction of hyperglycemia referred as T2DM in zebrafish. We used a comparison table in order to magnify the similarities and differences between the mentioned methods (Tables 1, 2). Also, scoring was suggested in order to choose the best model of hyperglycemia with the highest possible similarities to human among available ones (Tables 3, 4) based on the previous scoring principles for animal modeling of polycystic ovary syndrome as a metabolic disease (Tamadon et al., 2018). A verity of criteria is considered as laboratory workmanship and biological resemblance to human T2DM state. It is also possible to confront different scores for each model due to further studies on these criteria in the future.

**Table 1:** Comparison of non-genetic induced hyperglycemia models referred as T2DM in zebrafish.

**Model/method**	**Zebrafish life stage**	**Induction time**	**Pros**	**Cons**	**References**
Immersion alternatively in 2 and 0% glucose solution model of hyperglycemia	Adult (1–3 years old)	1 month	• Simple • Cheap • Retinopathy	• Needs continues alteration of solution • No investigation for insulin resistance property	Gleeson et al., 2007
Immersion in stepwise elevating glucose concentration model of hyperglycemia	Adult (1–3 years/5–11 months)	2 month/10 days	• Simple • Cheap • Insulin resistance • Anti-diabetic drug responsiveness	• No significant drawback	Connaughton et al., 2016
Chronic immersion in 110 mM glucose solution model of hyperglycemia	Adult stage (3–5 cm)	14 days	• Simple • Cheap • Fast^*^ • Persistent hyperglycemia • Anti-diabetic drug responsiveness • Elevated glycated proteins • Impaired insulin response	• 20% mortality • No investigation for insulin resistance property	Capiotti et al., 2014
Chronic immersion in 4% glucose solution model of hyperglycemia	Adult	28 days	• Simple • Cheap • Retinal vasculopathy	• Not suitable for female zebrafish • No investigation for insulin resistance property	Carnovali et al., 2016
Immersion in alternating 4 and 5% glucose solution model of hyperglycemia	Larva	5 days	• Simple • Cheap • Fast • Conserved retinopathic symptoms until adulthood	• High mortality • No investigation for insulin resistance property	Singh et al., 2019
Immersion in 130 mM glucose solution model of elevated hyperglycemia	Larva	3 days	• Simple • Cheap • Fast • Retinopathy	• No investigation for insulin resistance property	Jung et al., 2016
Obesity model of hyperglycemia	Adult (4–6 months)	6 weeks	• Simple • Cheap • Insulin resistance • Persistent hyperglycemia • Glucose intolerance • Increased β-cell mass	• Long time of induction	Zang et al., 2017
High fat diet (HFD) containing 1% egg yolk model of hyperglycemia	Adult	10 weeks	• Simple • Cheap • Insulin resistance	• Long time of induction	Meng et al., 2017
Bisphenol F induced model of hyperglycemia	Larva	2 days	• Simple • Cheap • Fast • Insulin resistance • Larval stage • Anti-diabetic drug responsiveness	• No significant changes to glucagon mRNA expressions	Zhao et al., 2018a
Bisphenol S induced model of hyperglycemia	Adult (9 months)	28 days	• Simple • Cheap • Fast	• Not elevated insulin levels	Zhao et al., 2018b
Combined high cholesterol diet (HCD) and high glucose (HG) environment model of hyperglycemia for larval zebrafish	Larva	10 days	• Simple • Cheap • Fast • Low mortality • Increased insulin • Increased glucagon • Increased total cholesterol	• No significant drawback	Wang et al., 2013
Combined high cholesterol diet (HCD) and high glucose (HG) environment model of hyperglycemia for adult zebrafish	Adult	19 days	• Simple • Cheap	• Not revealing insulin resistance symptoms	Wang et al., 2020

* *We considered an induction time of up to two weeks as fast induction*.

**Table 2:** Comparison of genetic induced hyperglycemia models referred as T2DM in zebrafish.

**Model/method**	**Zebrafish life stage**	**Induction time**	**Pros**	**Cons**	**References**
Tg (acta1: dnIGF1R-EGFP) transgenic line or zebrafish muscle insulin resistance (zMIR) model of hyperglycemia	Adult	No time	• Insulin resistance • Increased β-cell • Glucose intolerance	• Long period for exhibiting symptoms • Not elevated FBS without overfeeding • Not increased number of β cells in older group	Maddison et al., 2015
FOXN3 [Tg(fapb10a:foxn3,EGFP)z106 and Tg(fapb10a:FOXN3,EGFP)z107] models of hyperglycemia	Larva/adult	No time	• Stable hyperglycemia in both larval stage and adulthood	• No significant drawback	Karanth et al., 2016
K_ATP_ gain of function (K_ATP_-GOF) model of hyperglycemia	Larva/adult	No time	• Severely hyperglycemic • Increased gluconeogenesis • Decrease of glycolytic genes • Stable hyperglycemia in both larval stage and adulthood	• No significant drawback	Emfinger et al., 2019
Type 2 deiodinase (DIO2) knockout model of hyperglycemia	Adult	No time	• Insulin resistance • Similar enzymatic regulations as human	• Normoglycemia in older group • Not increased number of β and α cells	Houbrechts et al., 2019
Multiplex conditional mutagenesis model of hyperglycemia	Larva/adult	No time	• Insulin resistance • Post-prandial hyperglycemia	• No significant drawback	Maddison et al., 2015
Leptin receptor mutation model of hyperglycemia	Larva/adult	No time	• Increased insulin • Increased glucagon • Metformin responsiveness	• Not persistent hyperglycemia	Michel et al., 2016
• Pdx1 gene knockout model of hyperglycemia • PDX1 gene knockdown model of hyperglycemia	Larva/adult	No time	• Retinopathic symptoms • Post-prandial hyperglycemia • Susceptibility to high fat diet • Persistent hyperglycemia	• Not revealing insulin resistance, decreased β-cells	Kimmel et al., 2015; Wiggenhauser et al., 2020
MODY gene mutation model of hyperglycemia	Larva	No time	• Tolbutamide responsiveness	• No investigation for insulin resistance property	Mathews and Gustafsson, 2019
Aldh3a1 gene knockout model of hyperglycemia	Larva	No time	• 4-HNE elevation as HbA1c elevation • Retinopathy	• No investigation for insulin resistance property	Lou et al., 2020
G protein-coupled receptor 27 (Gpr27) knockout model of hyperglycemia	Larva	No time	• Post-prandial hyperglycemia • Insulin resistance • Metformin responsiveness	• No significant drawback	Nath et al., 2020
Glyoxalase1 (glo1^−/−^) knockout model of hyperglycemia	Larva/adult	No time	• Increased post-prandial glucose • Insulin resistance symptoms • Impaired glucose tolerance • Retinopathy • Persistent susceptibility to high fat diet from larval to adult stage	• Not persistent hyperglycemia • No direct insulin resistance assay (just p70-S6 kinase activation upregulation)	Lodd et al., 2019
Glucose transporter 12 (GLUT12) deficient model of hyperglycemia	Larva	No time	• Insulin resistance • Metformin responsiveness	• No significant drawback	Jiménez-Amilburu et al., 2015
Single insra or insrb knockout model of hyperglycemia	Larva	No time	• Post-prandial hyperglycemia • Insulin resistance	• No significant drawback	Yang et al., 2018

**Table 3:** Scoring of non-genetic induced hyperglycemia models referred as T2DM in zebrafish.

**Phenotype**	**Human disorder**	**Glucose induced models**						**Diet induced models**		**Chemical induced model**		**Hybrid models**	
Model/method	NA	Immersion alternatively in 2 and 0% glucose solution model of hyperglycemia	Immersion in stepwise elevating glucose concentration model of hyperglycemia	Chronic immersion in 110 mM glucose solution model of hyperglycemia	Chronic immersion in 4% glucose solution model of hyperglycemia	Immersion in alternating 4 and 5% glucose solution model of hyperglycemia	Immersion in 130 mM glucose solution model of elevated hyperglycemi	Obesity model of hyperglycemia	High fat diet (HFD) containing 1% egg yolk model of hyperglycemia	Bisphenol F induced model of hyperglycemia	Bisphenol S induced model of hyperglycemia	Combined high cholesterol diet (HCD) and high glucose (HG) environment model of hyperglycemia for larval zebrafish	Combined high cholesterol diet (HCD) and high glucose (HG) environment model of hyperglycemia for adult zebrafish
References	Cox and Edelman, 2009	Gleeson et al., 2007	Connaughton et al., 2016; Mohammadi et al., 2020	Capiotti et al., 2014	Carnovali et al., 2016	Singh et al., 2019	Jung et al., 2016	Zang et al., 2017	Meng et al., 2017	Zhao et al., 2018a	Zhao et al., 2018b	Wang et al., 2013	Wang et al., 2020
**SIMILARITIES TO HUMAN**													
Hyperglycemic outcome	*	*	*	*	*	*	*	*	*	*	*	*	*
Impaired GT^†^	*	#	#	#	#	#	#	*	#	#	#	#	#
HbA1c alternatives assay	*	#	#	*	#	#	#	#	#	#	#	#	#
Retinopathy	*	*	#	#	*	*	*	#	#	#	#	#	#
Insulin resistance	*	#	*	#	#	#	#	*	*	*	/	*	/
Anti-diabetic drug responsiveness	*	*	*	*	#	#	#	*	#	*	#	*	#
Stable hyperglycemia	*	#	#	*(7D)	#	#	#	*(1M)	#	#	#	#	#
Total score	7	3	3	4	2	2	2	5	2	3	0	3	0
**SUITABILITY AS A MODEL**													
Larval or embryonic stage	*	/	#	/	/	*	*	#	#	*	#	*	#
Adult stage	*	*	*	*	*	#	#	*	*	#	*	#	*
Induction time^‡^	^*^	^*^(1M)	^*^(2M/10D)	^*^(14D)	^*^(1M)	^*^(5D)	^*^(3D)	^*^(7D)	/(2.5M)	^*^(2D)	^*^(28D)	^*^(10d)	^*^(19d)
Total score	3	1	2	1	1	2	2	2	0	2	2	2	2
Model score	10	4	5	5	3	4	4	7	2	5	2	5	2

† *GT, Glucose tolerance; D, Day; M, Month*.

* *: Positive score = +1*.

/ *: Negative score = −1*.

# *: No information available = 0*.

‡ *Scoring of induction time: 0–30 days = +1; more than 30 days = −1*.

**Table 4:** Scoring of genetic induced hyperglycemia models referred as T2DM in zebrafish.

**Phenotype**	**Human disorder**	**Genetic models**												
Model/method	NA	Tg (acta1: dnIGF1R-EGFP) transgenic line or zebrafish muscle insulin resistance (zMIR) model of hyperglycemia	FOXN3 [Tg(fapb10a: foxn3,EGFP) z106 and Tg(fapb10a: FOXN3,EGFP) z107] models of hyperglycemia	K_ATP_ gain of function (K_ATP_-GOF) model of hyperglycemia	Type 2 deiodinase (DIO2) knockout model of hyperglycemia	Multiplex conditional mutagenesis model of hyperglycemia	Leptin receptor mutation model of hyperglycemia	Pdx1 gene knockout model of hyperglycemia PDX1 gene knockdown model of hyperglycemia	MODY gene mutation model of hyperglycemia	Aldh3a1 gene knockout model of hyperglycemia	G protein-coupled receptor 27 (Gpr27) knockout model of hyperglycemia	Glyoxalase1 (glo1^−/−^) knockout model of hyperglycemia	Glucose transporter 12 (GLUT12) deficient model of hyperglycemia	Single insra or insrb knockout model of hyperglycemia
References	Cox and Edelman, 2009	Maddison et al., 2015	Karanth et al., 2016	Emfinger et al., 2019	Houbrechts et al., 2019	Yin et al., 2015	Michel et al., 2016	Kimmel et al., 2015; Wiggenhauser et al., 2020	Mathews and Gustafsson, 2019	Lou et al., 2020	Nath et al., 2020	Lodd et al., 2019	Jiménez-Amilburu et al., 2015	Yang et al., 2018
**SIMILARITIES TO HUMAN**														
Hyperglycemic outcome	*	*	*	*	*	*	*	*	*	*	*	*	*	*
Impaired GT^†^	*	*	#	#	#	*	/	*	#	#	*	*	#	*
HbA1c alternatives assay	*	#	#	#	#	#	#	#	#	*	#	*	#	#
Retinopathy	*	#	#	#	#	*	#	*	#	*	#	*	#	#
Insulin resistance	*	*	#	#	*	*	*	#	#	#	*	*	*	*
Anti-diabetic drug responsiveness	*	#	#	#	#	#	*	*	*	#	*	#	*	#
Stable hyperglycemia	*	*	*	*	*	*	/	*	#	#	#	*	#	#
Total score	7	4	2	2	3	5	1	5	2	3	4	6	3	3
**SUITABILITY AS A MODEL**														
Larval or embryonic stage	*	#	*	*	#	*	*	*	*	*	*	*	*	*
Adult stage	*	*	*	*	*	#	*	*	#	#	#	*	#	#
Induction time^‡^	*	^*^(0)	^*^(0)	^*^(0)	^*^(0)	^*^(0)	^*^(0)	^*^(0)	^*^(0)	^*^(0)	^*^(0)	^*^(0)	^*^(0)	^*^(0)
Total score	3	2	3	3	2	2	3	3	2	2	2	*	2	2
Model score	10	6	5	5	5	7	4	8	4	5	6	9	5	5

† *GT, Glucose tolerance; D, Day; M, Month*.

* *: Positive score = +1*.

/ *: Negative score = −1*.

# *: No information available = 0*.

‡ *Scoring of induction time: 0–30 days = +1; more than 30 days = −1*.

## Materials and Methods

### Software, Databases, and Search Queries

We investigated four databases including Google scholar, PubMed/Medline, Microsoft academic, and Scopus. We used the following search query in titles for Google scholar, Microsoft academic, and Scopus databases: “(zebrafish OR danio) AND (diabetes OR diabetic OR hyperglycemia OR hyperglycemic OR glucose OR pancreas OR pancreatic OR insulin OR MODY).” For the PubMed/Medline database, we used “(zebrafish [Title/Abstract] OR danio [Title/Abstract]) AND (diabetes [Title/Abstract] OR diabetic [Title/Abstract] OR hyperglycemia [Title/Abstract] OR hyperglycemic [Title/Abstract])” search query to screen all related articles by title and abstract, simultaneously. Google scholar, Scopus, and Microsoft academic searches were performed using the Publish or Perish Windows software. Then, the PubMed/Medline results were added and duplicate results were removed using the Mendeley Desktop software (1,220 papers were found in general). We screened the remaining results by title and abstract to investigate available hyperglycemia models referred as T2DM in zebrafish. Publication date was not restricted.

### Inclusion and Exclusion Strategy

**Figure 1:**
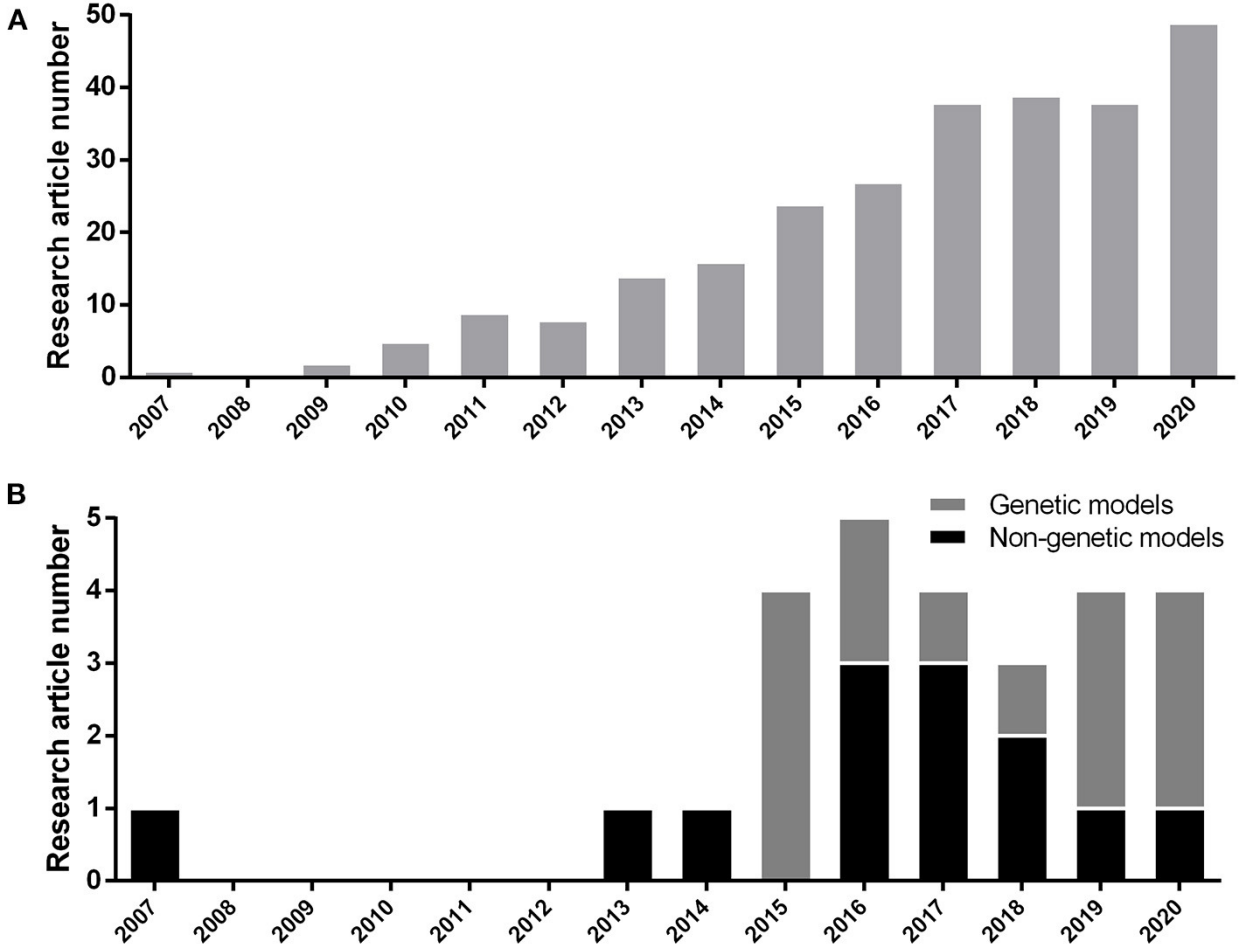
**(A)** Number of research article publications by year to demonstrate the growing interest of researchers by hyperglycemic model of zebrafish (*Danio rerio*) based on the PubMed/Medline database. **(B)** Development of new genetic or non-genetic hyperglycemic model of zebrafish (*Danio rerio*) by year.

We included the results of every study that was consistent with hyperglycemic outcome (whether fasting blood glucose elevation, post-high content diet feeding blood glucose elevation, post-prandial glucose elevation, or whole larval glucose elevation) in the first place and have utilized one additional approval method which will be explained in detail further (section Methods of Model Verification). Selected articles are based on the induction of chronic hyperglycemia as T2DM occurs in human and without considering any publishing time limitations. We considered MODY studies too, because they manifest T2DM in the early stages of human life by involving well-known specific genes. Disruption of these genes increase the chance of T2DM occurrence. Therefore, they can be suitable subjects to study T2DM pathophysiology in animal models, too. We excluded every study with relation to T1DM induction methods. These studies consist of alloxan or streptozotocin (STZ) focused means of induction. Acute or non-persistent hyperglycemic states, such as glucose injection were disqualified too. During 2007 to 2020, based on the PubMed/Medline database, 270 research articles have been published on hyperglycemic model of zebrafish (Figure 1A). We selected 26 methodological papers of hyperglycemia referred as T2DM induction to be reviewed in our study, systematically (Figure 1B).

### Scoring Rationales and Principles

The scoring system was obtained from the previous study by Tamadon et al. (2018). All the major diabetic outcomes in the proposed models are included and compared to human diabetic state, respectively. In the scoring tables (Tables 3, 4), any similarity between zebrafish and human diabetic state is graded as one positive score (marked as ^*^ in the table), any inconsistency with human diabetic state is graded as one negative score (marked as / in the table), and any unavailable data for the proposed factor is considered as zero or neutral (marked as # in the table). Finally, all the scores of a single model are added up and the final score of the model is generated.

For a better comprehension and model selection for further studies, we separated all the scoring factors into two main categories; firstly, as the similarities to human and secondly, as the suitability of the model. All the factors for human scoring are regarded as one positive score and all the suitability factors are considered to be met by the perfect model (score of 10). Therefore, the highest grade is achieved in the first column of the table (human disorder). All the verified models are included in the table and evaluated in proportion to the ideal model (first column).

All available models can be categorized as larval or adult stage zebrafish models. It wholly depends on the researcher's intention to find the most suitable model considering their own purposes, research theme, limitations, and circumstances. We considered a one plus score for the availability of the model in both zebrafish life stages. As we can see, some models can be utilized in both larval and adult stages, receiving a higher score. Induction time can be a crucial factor. Therefore, we considered a positive score for faster induction time (<1 month) due to faster results, higher efficiency of the model, and workmanship and deducted a score for more induction time. As we can see, genetic models are ahead in terms of time saving factor (0 days for induction).

## Methods/Models of Inducing Hyperglycemia in Zebrafish

### Glucose Based Methods of Inducing Hyperglycemia

Due to the ease of induction and cheapness, glucose-based induction is favorable. There are different protocols which have been developed based on matter of time, life stage, and glucose concentration. We can consider this approach of T2DM induction in zebrafish as the first approach ever, which is modified and has evolved over years.

#### Immersion Alternatively in 2% and 0% Glucose Solution Model of Hyperglycemia

Gleeson et al. (2007) introduced a primary method of inducing T2DM in zebrafish. In this method, zebrafish were alternately exposed to 2% glucose and 0% glucose solution for 30 days. Common diabetes symptoms, such as retinopathy and increased fasting blood glucose (FBS) levels were observed afterwards. The FBS for control groups of wild-type zebrafish were measured 74 ± 8.5 and 89 ± 10.6 mg/dL for AB type. The FBS passed 200 mg/dL (three times more the normal state) after 30 days.

#### Immersion in Stepwise Elevating Glucose Concentration Model of Hyperglycemia

Connaughton et al. (2016) exhibited that the previous method is age-dependent. In this article, two groups of zebrafish (1–3 years old) and (4–11 months old) were investigated. Initially, both groups were immersed in 2 and 0% alternating glucose solution for 2 months (as mentioned in the Immersion Alternatively in 2 and 0% Glucose Solution Model of Hyperglycemia method). In the older group, high blood glucose levels were observed for the entire 2 months, but the younger fish group temporarily gained higher glucose levels (up to first month) and then adopted to the new environment and returned to the normoglycemic state. To solve this challenge, they modified the protocol and exerted an alternative method so that the concentration of glucose was elevating within the progress of induction stages. Initially, they incubated the younger zebrafish in 1% glucose solutions for 2 weeks. They increased glucose concentration up to 2% in the next 2 weeks and after that, incubated the young adult zebrafish in 3% glucose solution for the next month. The younger group of zebrafish gained a stable hyperglycemia state for the entire 2 months of induction.

In another study by Mohammadi et al. (2020), they used the same approach in a much shorter time. They incubated the adult zebrafish for 4 days in 50 mM glucose solution, followed by 100 mM glucose concentration immersion for the next 3 days. Finally, they elevated the glucose concentration to 200 mM for the last 3 days (10 days total). They showed that their approach is in compliance with previous study by revealing higher glucose concentrations and being responsive to metformin as an anti-diabetic drug (Mohammadi et al., 2020).

#### Chronic Immersion in 110 mM Glucose Solution Model of Hyperglycemia

Capiotti et al. (2014) introduced another method of inducing diabetic symptoms. Initially, three concentrations of 55, 110, and 166 mM of glucose solution were investigated. Due to the lower mortality rate of fish compared to the other two concentrations, they chose 110 mM concentrations for induction. In a 5-l tank containing 110 mM glucose, 15 zebrafish were incubated for 14 days. After 14 days, blood glucose levels increased up to four or five times in comparison to the control group. To assess the stability of this process, the zebrafish were placed in fresh water for 7 days. Measurements showed that blood glucose was still higher than the control group. By assessing fructosamine as an indicator of non-enzymatic glycation of proteins from the eyes of the zebrafish, they noticed elevated levels of fructosamine in the test group and even in the group that had been isolated for 7 days in fresh water, which confirms the reliability of the procedure. Therapies with anti-diabetic drugs, such as glimepiride and metformin were applied, which represent two different mechanisms of therapeutic. Short induction time as well as stability of hyperglycemic status are both prominent advantages of this method (Capiotti et al., 2014).

#### Chronic Immersion in 4% Glucose Solution Model of Hyperglycemia

Carnovali et al. (2016) used a different approach of chronic immersion in glucose solution. In this study, 4% glucose solution was used for 28 continuous days in order to induce hyperglycemia. This method also revealed insulin resistance and retinal vasculopathy as a result.

#### Immersion in Alternating 4% and 5% Glucose Solution Model of Hyperglycemia

In order to produce hyperglycemic state, Singh et al. (2019) used 4 and 5% alternating glucose solution individually from 3 h post-fertilization (hpf) to 5 days' post-fertilization (dpf). This means that the induction resulted in an increased total free glucose and retinopathic symptoms that were conserved up to adulthood. They also suggested that this induction method mimics fetus condition in mothers with pre-existing and gestational diabetes. It is worth mentioning that alternating immersion of zebrafish in 4 and 5% glucose solution caused 28.4 and 37.5% of mortality rate, accordingly.

#### Immersion in 130 mM Glucose Solution Model of Elevated Hyperglycemia

Another glucose based approach of hyperglycemia induction that Jung et al. (2016) utilized was the immersion in 130 mM glucose solution from 3 to 6 dpf. The approach showed to be effective by the elevation of the whole larval glucose and showing retinopathic symptoms, such as thickening of the vascular basement membrane and increased vascular permeability (Robinson et al., 2012).

### Diet Based Methods of Inducing Hyperglycemia

Improper diet, overweight, or obesity are proved to be major reasons of diabetes occurrence (Hu et al., 2001). Zebrafish is considered to be a suitable model due to possessing major organs with high resemblance to human counterparts, such as kidney, pancreas, adipose tissue, and skeletal muscle (Schlegel and Stainier, 2007). This method can be considered as one of the most effective and most accessible ways to exhibit hyperglycemic state in zebrafish. It is worth mentioning diet based method of producing T2DM in zebrafish was inspired from rodents obesity and insulin resistance model (Tobey et al., 1982; Surwit et al., 1988).

#### Obesity Model of Hyperglycemia

Interestingly, 90% of people with T2DM are overweight or obese. Therefore, there would be observations of diabetic symptoms by inducing obesity (Scherer, 2006). Zebrafish has exhibited conserved pathophysiological pathways with mammalian obesity (Oka et al., 2010). In a study by Zang et al. (2017), these principles were utilized to produce hyperglycemic condition in zebrafish. Zebrafish were fed Otohime B2 six times a day for 8 weeks (408 calories for the test group and 68 calories for the control group). FBS level showed a significant increase compared to the control group by the first week and kept the hyperglycemic state constant throughout the procedure. Up to 4 weeks after stopping treatment, despite a slight decrease, FBS remained higher than in the control group. They also used the anti-diabetic drug responsiveness test, glucose tolerance test, and deep RNA sequencing in order to verify this model. This method can be considered as the most available approach to study diabetes utilizing zebrafish.

#### High Fat Diet (HFD) Containing 1% Egg Yolk Model of Hyperglycemia

In another study by Meng et al. (2017), they utilized HFD consisted of brine shrimp and 1% egg yolk for 10 weeks, and the results were consistent with the previous obesity induced hyperglycemia method. They revealed that insulin receptor substrate 2 (IRS2) and glucose transporter 2 (GLUT2) were significantly decreased in livers of HFD-treated zebrafish and insulin levels were elevated in the muscle and liver tissue significantly suggesting insulin resistance induction.

### Chemical Based Methods of Inducing Hyperglycemia

Etiology of diabetes has been pursued over years. Different chemicals that may be diabetogenic have surrounded us by different doses or exposure period. Chemicals, such as alloxan and streptozotocin are widely used to induce diabetes in rodents (Engel et al., 2019). In two studies, effects of bisphenol compounds as common chemicals on glucose metabolism of zebrafish have been investigated.

#### Bisphenol F Induced Model of Hyperglycemia

Zhao et al. (2018a) showed the effects of bisphenol F on glucose homeostasis in zebrafish larvae. Background of this study had exhibited insulin resistance, decreased glucose tolerance, and higher levels of plasma insulin, triglycerides, and leptin in mice. In this study, larval zebrafish was exposed to different doses of bisphenol F, but only 10 and 100 μg/L concentrations showed diabetic symptoms like increased levels of insulin and decreased transcription levels of genes which encode insulin receptor substrate. They also showed an increase in mRNA transcription of key gluconeogenesis enzymes. Insulin levels increased and transcription levels of genes encoding insulin receptor substrates decreased significantly suggesting insulin resistance. All these signs imply to look at this model as a qualified approach.

#### Bisphenol S Induced Model of Hyperglycemia

Another study by Zhao et al. (2018b) revealed that bisphenol S also generates a diabetic state. Exposure to 1 and 10 μg/L of bisphenol S significantly increased FBS levels. In spite of insulin levels reduction, gluconeogenesis and glycogenolysis in the liver were promoted, glycogen production in the liver and muscle and glycolysis in the muscle were inhibited (Zhao et al., 2018b). These clues show that bisphenol S is likely to be a diabetogenic agent in zebrafish.

### Hybrid Based Methods of Inducing Hyperglycemia

It is also recommended to use a combination of available methods in order to develop a more consistent condition to human diabetes. This approach was also inspired by rodents' studies. Combination of techniques can be promising to develop solid models (Kurup and Bhonde, 2000; Pinhas-Hamiel and Zeitler, 2005; Zhang et al., 2008).

#### Combined High Cholesterol Diet (HCD) and High Glucose (HG) Environment Model of Hyperglycemia for Larval Zebrafish

Wang et al. (2013) developed a hybrid method so that a HCD (10% cholesterol) was used and fish were exposed to HG (2% glucose) solution, simultaneously similar to some methods used for mice. This method created a manifestation of diabetes by causing vascular disorders. Wild type zebrafish and various transgenic lines have been used in this study. The levels of insulin, glucagon, glucose, triglyceride, and cholesterol increased significantly. The model was responsive to anti-diabetic drugs, such as metformin and pioglitazone (Wang et al., 2013).

#### Combined High Cholesterol Diet (HCD) and High Glucose (HG) Environment Model of Hyperglycemia for Adult Zebrafish

In another study, Wang et al. (2020) revealed that exposure to 2% glucose solution and consuming 10% cholesterol diet for 19 days induces hyperglycemic state in adult zebrafish. They measured various parameters to achieve a better comprehension of hyperglycemic state related inflammation and apoptosis. While glucose and glucagon levels increased considerably, expression increase of insulin and insulin receptor mRNAs were not significant. These studies reveal that the hybrid approach (HCD-HG) of producing hyperglycemic state in zebrafish exhibits symptoms that are more accurate while exploited in earlier stages of zebrafish life.

### Genetic Based Methods of Inducing Hyperglycemia

Genetic manipulation of animals is widely used for a verity of scenarios. Using genetic disease modeling in zebrafish is getting more and more desirable by researchers (Phillips and Westerfield, 2019). Techniques, such as Tol2 (Kwan et al., 2007), clustered regularly interspersed short palindromic repeats (CRISPR) (Cong et al., 2013; Qi et al., 2013), zinc finger nuclease (ZFN) (Doyon et al., 2008), morpholino injection (Partridge et al., 1996), and TALEN (Miller et al., 2011) based methods are popular in generation of a hyperglycemic state.

#### Zebrafish Tol2 Models of T2DM

##### Tg (acta1: dnIGF1R-EGFP) Transgenic Line or Zebrafish Muscle Insulin Resistance (zMIR) Model of Hyperglycemia

Maddison et al. (2015) made zebrafish insulin resistant by expressing dominant-negative insulin growth factor (IGF) in skeletal muscle that led to β-cell dysfunction, too. These zebrafish showed insulin-signaling disorders, which is similar to the symptoms of T2DM in humans. It was initially associated with an increase in the number of pancreatic β-cells up to the first 6 months (in order to compensate for insulin resistance), which maintained a normal blood glucose. Then, the number of β-cells decreased and got equal to the control group at the end of the first year. Overfeeding was followed by glucose intolerance and an increase in FBS, which indicated pancreatic β-cell vulnerability similar to the symptoms of T2DM. Glucose intolerance progress was slow and there was no change in FBS without over nutrition (Maddison et al., 2015). Long time to exhibit symptoms is the major drawback of this method, but remarkable stability and similarity to the human model are major advantages.

##### FOXN3 [Tg(fapb10a:foxn3,EGFP)z106 and Tg(fapb10a:FOXN3,EGFP)z107] Models of Hyperglycemia

Karanth et al. (2016) could reach higher blood glucose levels by overexpressing a specific section of the first intron in FOXN3 gene by Tol2 transposon-mediated transgenesis. The gene carrier was determined to have a higher hepatic expression and downregulate in fasting conditions. Overexpressing of the named gene led to an elevated whole larval free glucose and higher adult FBS by hepatic gluconeogenic gene expression and suppression of *mycb* (human MYC ortholog, which is the expression stimulator of glucose consumer enzymes). Stable hyperglycemia in both larval stage and adulthood is a significant characteristic of this model.

##### K_ATP_ Gain of Function (K_ATP_-GOF) Model of Hyperglycemia

K_ATP_ channels in β-cells control membrane excitability by glucose stimulated insulin secretion (Ashcroft and Rorsman, 1990). The channel is expressed in β-cells of zebrafish with similar functionality to their mammalian orthologs as well (Emfinger et al., 2017). In the following study, Emfinger et al. (2019) produced higher blood glucose levels by making gain-of-function mutations in ATP dependent potassium channels of islet β-cells of zebrafish. These fish showed a slow growth and deficient glucose-induced calcium response, too. They used Tol2 transposase insertion to express cytosolic gCAMP6s.

#### Zebrafish Zinc Finger Nuclease (ZFN) Models of T2DM

##### Type 2 Deiodinase (DIO2) Knockout Model of Hyperglycemia

In previous studies, it has been shown that DIO2 polymorphism is correlated with an increased FBS and insulin levels, insulin resistance, and susceptibility to diabetes (Canani et al., 2005; Dora et al., 2010). They developed two group of zebrafish DIO2 knockdown. The first and the second group were 6–9 and 18–24 months old, respectively. The knockout made the younger group hyperglycemic up to 1-year-old, accompanied with an increase in insulin and glucagon levels and reduction of insulin receptors on skeletal muscle. Older group showed a reduction in the glucagon receptors, glucose transporters, and G6PD. and were normoglycemic unlike the younger group (Houbrechts et al., 2019).

#### Zebrafish CRISPR Models of Hyperglycemia

##### Multiplex Conditional Mutagenesis Model of Hyperglycemia

Yin et al. (2015) produced hypopigmentation and defects of glucose hemostasis by the inactivation of insulin receptors (insra and insrb) and tyrosinase (tyr) genes, simultaneously. They also targeted ascl1 gene by sgRNAs injection to retinas and revealed a decrease in the number of proliferative cells after exposure to constant, high-intensity light. This model showed a lower FBS, insulin resistance, and post-prandial hyperglycemia eventually.

##### Leptin Receptor Mutation Model of Hyperglycemia

Leptin is a primary adipostatic factor produced by adipocytes in proportion to adipose mass in mammals. Lack of leptin receptors in mice produces obesity, hyperphagia, hyperinsulinemia, and diabetes (Halaas et al., 1995). Unlike mammals that express leptin receptor in adipose tissue mostly, fish highest expression takes place in the brain, white muscle, liver, and ovaries than adipose tissue (Kurokawa et al., 2005; Rønnestad et al., 2010). Proposed model by Michel et al. (2016) exhibited how leptin receptor signaling effects glucose homeostasis in zebrafish. They showed that mutagenesis of lepr and lepa, but not lepb genes exhibited increased β-cell number and hyperinsulinemia, hyperglucagonemia, and diabetes symptoms in larval stages. They also investigated the possible effects in adulthood but did not find an elevated FBS.

##### Pdx1 Gene Knockout Model of Hyperglycemia

Pancreatic and duodenal homeobox 1 (Pdx1) is essential for pancreas development and β-cell function (Fujimoto et al., 2009). Homozygous absence of PDX1 gene alleles is the indicator of early onset of T2DM (MODY4), but partial functionality of PDX1 gene increases the risk of T2DM occurrence (Staffers et al., 1997; Macfarlane et al., 1999; Weng et al., 2001).

In a study, it has been shown that CRISPR/Cas9-mediated gene knockout of pdx1 gene in zebrafish is followed by retinopathic symptoms and post-prandial hyperglycemia (Wiggenhauser et al., 2020). This study is considered in the same category within Tables 2, 4 as the PDX1 gene knockdown model of hyperglycemia.

##### MODY Gene Mutation Model of Hyperglycemia

In a study by Mathews and Gustafsson (2019), the effect of common anti-diabetic drug tolbutamide on five major orthologs of human MODY related genes (gck, hnf1a, hnf1ba, hnf1bb, and pdx1) in zebrafish was assessed. They showed that tolbutamide intervention ameliorated induced a hyperglycemic state significantly. A mixture of high cholesterol diet (4%) and immersion in glucose solution (3%) from 5 to 9 dpf were performed to challenge zebrafish larvae metabolically. More investigations on these mutations solely or in combination are required to assess T2DM characteristics thoroughly.

##### Aldh3a1 Gene Knockout Model of Hyperglycemia

Methylglyoxal (MG) is the major precursor of advanced glycation end products (AGEs) (Thornalley, 2008) which is degraded by glyoxalase system existing in the cytosol of mammalian cells (Rabbani and Thornalley, 2011). MG can also be degraded by aldo-keto-reductase and aldehyde dehydrogenase (ALDH) (Vander Jagt and Hunsaker, 2003). ALDH 3 families, member A1 (Aldh3a1) generally oxidizes lipid peroxidation aldehydes to carboxylic acids (Pappa et al., 2005). The gene is available in human, mouse, and zebrafish. In zebrafish, the gene shares 61.6% to human ALDH3A1 (Lou et al., 2020). Lou et al. (2020) revealed that aldh3a1^−/−^ knockout in zebrafish is accompanied with an increased whole larval glucose, retinal vasodilatory alterations, and generally impaired glucose metabolism. They also showed a correlation between HbA1c increase and 4-hydroxynonenal (4-HNE). This specific mutant hyperglycemic state was amplified by pdx1 gene silenced mutant and achieved a higher level of free larval glucose accordingly (Lou et al., 2020).

##### G Protein-Coupled Receptor 27 (Gpr27) Knockout Model of Hyperglycemia

Gpr family is well-conserved among vertebrates (Matsumoto et al., 2000). Among the family members, Gpr27 has shown to be expressed mostly in brain tissue and in several tissues like pancreas, skeletal muscle, and adipose tissue (Lonsdale et al., 2013). It has been revealed that the knockdown of Gpr27 in mouse pancreas caused a declination in insulin promotor activity (Ku et al., 2012). Knock out of the mentioned gene in zebrafish showed consistent results with the mammalian model. The fish did not show high FBS with normal diet, but a significant increase with a high fat diet, as we see in human T2DM state. The fish were also resistant to insulin injection, suggesting insulin resistance condition and a solid model for subsequent studies of T2DM (Nath et al., 2020).

##### Glyoxalase1 (glo1^−/−^) Knockout Model of Hyperglycemia

In a study by Lodd et al. (2019), permanent knockout of glyoxalase 1 using CRISPR/Cas9 was performed in order to evaluate long-term effects of the mentioned gene on glucose metabolism and the vascular system in juvenile and adult stages of zebrafish. The knockout model showed increased MG concentrations in the tissue, temporary increased post-prandial glucose, insulin resistance symptoms (p70-S6 kinase activation upregulation), impaired glucose tolerance, and increased retinal blood vessel sprouting after overfeeding suggesting retinopathic susceptibility. The remarkable point of the model is that (glo1^−/−^) knockout zebrafish are normoglycemic in the fasting condition but also show slower glucose homeostasis after feeding (Lodd et al., 2019). Although the model does not show persistent hyperglycemia and is dependent on overfeeding to show some symptoms, it can potentially be considered as the most well-studied model to study T2DM in zebrafish so far.

#### Morpholino Injection Models of Hyperglycemia

##### PDX1 Gene Knockdown Model of Hyperglycemia

Kimmel et al. (2015) could achieve a persistent hyperglycemic state in zebrafish by pdx1 gene knockdown utilizing morpholino injection. The model showed reduced β-cell number and insulin levels resulting in a persistent hyperglycemic state. Hyperglycemic state was consistent before feeding started all the way to adult stage. β-cells susceptibility of pdx1 mutants was revealed while larvae were exposed to a high fat diet. Despite revealing persistent hyperglycemia as a notable benefit, not revealing insulin resistance and decrease of β-cell numbers may incline the model to T1DM rather than T2DM, generally. This study is considered to be in the same category within following tables as the Pdx1 gene knockout model of hyperglycemia.

##### Glucose Transporter 12 (GLUT12) Deficient Model of Hyperglycemia

GLUT12 is generally expressed in insulin-sensitive tissues like heart, skeletal muscle, and adipose tissue. In a study by Jiménez-Amilburu et al. (2015), it is has been shown that the gene is on the same chromosme and mainly expressed in the same tissues as human and zebrafish preserves the same functionality of GLUT12 as human does. Morpholino knockdown of GLUT12 lead to hyperglycemia and insulin restance related genes disruption. Larvae showed a higher expression of GLUT12 in response to insulin injection and zebrafish larvae were also responsive to anti-diabetic drug, metformin.

#### TALEN Mediated Models of Zebrafish Hyperglycemia

##### Single Insra or Insrb Knockout Model of Hyperglycemia

The insr is considered as a member of the tyrosine kinase receptor subfamily (De Meyts et al., 2008). It has been shown that the targeted disruption of insr gene is followed by neonatal lethality in mice. A 1,000-fold increase in glucose levels after normal feeding showed the seriousness of insr gene in glucose metabolism (Joshi et al., 1996). Unlike humans, zebrafish owns a duplication of insr gene generally referred as insra and insrb. Both revealed to be homologous to human insr gene by 68.3 and 65.1% similarity (Toyoshima et al., 2008). A double knockout of insra and insrb yielded zebrafish death by 16 dpf that was a confirmation to the mouse model study (Yang et al., 2017).

In a subsequent study by Yang et al. (2018) it has been shown that single insra or insrb knockout models of zebrafish showed higher blood glucose, post-prandial hyperglycemia, and increased weight gaining compared to the control group, while being viable unlike the double knockout model. These findings suggest this model to be an interesting model of diabetes research in zebrafish.

## Methods of Model Verification

A verity of procedures is performed in order to verify hyperglycemic models as T2DM. Most common and acceptable ones are listed below.

### Hyperglycemic Outcome

In human, high fasting blood glucose is usually the first indicator of T2DM existence and a clue to further investigations. In the reviewed studies, fasting blood glucose elevation (Gleeson et al., 2007; Connaughton et al., 2016), post-high content diet feeding blood glucose elevation (Yang et al., 2018), post-prandial glucose elevation (Nath et al., 2020), or whole larval glucose elevation (Karanth et al., 2016) are reported as the primary indicator of T2DM existence possibility.

Studies of zebrafish diabetes model can be categorized as adult and larval stage studies. Unlike adult stage, larval stage study of diabetes is limited due to the lack of blood. The suggested solution is homogenizing the larvae and using colorimetric assays to determine whole larval glucose. For adult stage, there are different methods of blood sampling and measuring glucose including tail ablation (Gleeson et al., 2007), decapitation (Eames et al., 2010), blood collection from dorsal aorta (Jagadeeswaran et al., 1999), and cardiac puncture (Moss et al., 2009). Procedure of repetitive non-lethal blood sampling is developed, which sure is a more ethical way of performing such tests (Zang et al., 2013).

### Insulin Resistance Test

Insulin resistance is considered as one of the main symptoms of T2DM. In order to show insulin resistance, different approaches were suggested, such as measuring insulin (Meng et al., 2017), measuring insulin receptors (Houbrechts et al., 2019), measuring MG (Moraru et al., 2018), measuring related mediators (Nath et al., 2020), and insulin injection (Connaughton et al., 2016).

### Glucose Tolerance Test (GTT)

This test is based on glucose intolerance and can be used as a confirmation test of T2DM model for zebrafish. This test is done in both intraperitoneal and oral forms for adult zebrafish (Kinkel et al., 2010). Due to the lack of blood in the larval stages of zebrafish life, gene expression assays are utilized to assess related genes, such as GLUT (Jiménez-Amilburu et al., 2015) or post-prandial hyperglycemia (Lodd et al., 2019) as an indicator of T2DM.

### HbA1c Alternatives Assay

HbA1c in human is considered as a precise marker of diabetes. It is a glycated form of hemoglobin that increases in a hyperglycemic state. Therefore, it can be used as an important verification of diabetes existence (Mostafa et al., 2010). It is possible to extract fructosamine from the eyes of zebrafish and consider its increase as an upregulation of non-enzymatic glycation of proteins and diabetes (Armbruster, 1987; Capiotti et al., 2014). Other compounds, such as 4-hydroxynonenal (4-HNE) (Lou et al., 2020) and MG (Lodd et al., 2019) are proposed as indicators, too.

### Anti-diabetic Drug Responsiveness Test

Another method to verify the generated model is to use anti-diabetic drug assessing. This method ensures the existence of the same metabolic pathways as humans, which through drugs can be effective. Glimepiride, metformin (Capiotti et al., 2014), tolbutamide (Mathews and Gustafsson, 2019), and pioglitazone (Wang et al., 1998) are proposed in this segment and can effect glucose metabolism by different mechanisms.

### Withdrawal of Inducer Assay/Maintaining Stable Hyperglycemia Overtime

Due to different mechanisms of compatibility to a newer condition, inducing persistent hyperglycemic state is challenging in zebrafish. In different studies, after getting a hyperglycemic state, the inducer is removed and zebrafish is monitored to see how long it takes to get normoglycemic again. The longer the period, the better the induction method (Capiotti et al., 2014). Also, in some genetic studies like 2.5.1.2 method (Karanth et al., 2016) and 2.5.1.3 method (Emfinger et al., 2019), it has been shown that the proposed model can maintain an inert hyperglycemia over long periods of time (both larval and adult stage), promoting these mutant lines to valuable models of hyperglycemic state researches.

### Gene Expression Assay/Glucose Homeostasis Enzyme Assay

Glucose homeostasis includes an extensive number of enzymes and factors. It is possible to confirm a model by key enzymes involving related pathways. Insulin signaling pathway-related genes, glycolysis-related genes, gluconeogenesis-related genes (Yang et al., 2018), insulin resistance related genes, and glucose transporters (Jiménez-Amilburu et al., 2015) are proposed to assess and interpret the yielded state thoroughly.

### High Content Diet/High Content Water Immersion Challenge

In some studies, it has been suggested to challenge zebrafish after the induction period with food intake or immersion in high content water and determine if there is post-prandial diabetic like symptoms in comparison to a control group. High content diets, such as high calorie diet (Maddison et al., 2015), high carbohydrate diet (Yang et al., 2018), high fat diet (high cholesterol diet) (Kimmel et al., 2015; Nath et al., 2020), high cholesterol water immersion (Yin et al., 2015), or a combination of these (Mathews and Gustafsson, 2019) are proposed to see how much resemblance to human diabetic state is produced as a result of a hyperglycemia inducer agent.

## Discussion

Scientists have preferred rodents for diabetes modeling so far. Despite their physiological and biochemical advantages, they also have limitations. The zebrafish model has recently attracted much attention for metabolic and genetic studies. Also for diabetes studies, it represents major similarities to humans. Different approaches were suggested in order to study diabetes. Different approaches for induction of T2DM have been developed including glucose-induced models, diet based methods, chemical methods, genetic methods, and hybrid methods. In 2007, a study of T2DM by modeling in zebrafish was officially initiated (Gleeson et al., 2007). The idea of using glucose in the article was followed by next studies, too. These studies suggest practical and feasible models for studying genetic and physiological pathways involving the occurrence of diabetic symptoms. Obesity induced diabetes is favorable for studies of lipid metabolism in correlation of diabetic syndrome. Combination of these two approaches comes to another model that is proposed to study vascular disorders. The model represents high similarity to human disorders by being responsive to anti-diabetic compounds.

Chemicals penetrate to human body unintentionally and create a variety of side effects. Bisphenol, as a highly usable industrial chemical, was confirmed to produce diabetic symptoms in mice. In zebrafish, two different studies in larval and adult stages imply that this chemical can be used as a suitable diabetogenic reagent. They were also successful to target diabetes related genes in zebrafish and produce persistent hyperglycemia as well. Non-genetic means of hyperglycemia induction can be time consuming in comparison to genetic means. In contrast, they can be easily attained, need far less expensive facility to produce and maintain, and require less expertise to induce. Overall, the best method by our developed scoring table between non-genetic-induced zebrafish model of T2DM was the obesity model of hyperglycemia (Zang et al., 2017).

Initially, it is worth noting that genetic models gain higher scores on average. In this segment, although PDX1 gene knockdown/knockout (Kimmel et al., 2015; Wiggenhauser et al., 2020) gains a high score of eight, this does not reveal insulin resistance symptoms, making this model better suited for studying T1DM (Saberzadeh-Ardestani et al., 2018). The overall best model also lies among the genetic models. The 2.5.3.7 model by Lodd et al. (2019) is a well-studied mutant line of zebrafish for further study of T2DM. Of course, the model is accompanied with major drawbacks like being dependent on overfeeding to manifest T2DM symptoms or not assessing insulin resistance directly, however, it is the best evaluated model of hyperglycemia referred as T2DM in zebrafish overall.

Moreover, this systematic review article introduced eight procedures that have been exploited in order to verify hyperglycemic models as T2DM. Considering using larval or adult model, various evaluation tests have been used in T2DM zebrafish models. Furthermore, for selecting the appropriate tests, the types of the models of T2DM, non-genetic-induced zebrafish model, and/or genetic-induced zebrafish model must be considered. These methods are within good and bad prospects. They should be utilized by consideration of study purposes, facility, and related professional experiences of researchers.

## Future Insights

All the efforts are aimed to find the absolute pathophysiology of diabetes mellitus and propose a definitive cure. Animal modeling is still the leading resembling platform to study diseases and cures. As we have seen in this systematic review, zebrafish has served as a valuable model due to its exceptional characteristics. Different models are summoned, reviewed, and compared with each other and human as well. All models reveal strengths and weaknesses. It is possible to design and score new models of zebrafish with the current scoring system. Due to the development of new tools for genetic manipulation and disease induction, it is expected from the zebrafish model of T2DM to play a key role in attaining definitive cure and accelerate the research process considerably. In addition, integration of the current modeling of T2DM with new technologies, such as microfluidics by using zebrafish embryo, fish-on-a-chip, computational drug screening, and three-dimensional image analysis will facilitate for finding therapeutic approaches for this syndrome.

## Author Contributions

AS, MR, AK, YT, and AT conceived and designed the format of the manuscript. AS, MR, and AK drafted and edited the manuscript. AS and AT drew the tables. All authors reviewed the manuscript, contributed to the critical reading and discussion of the manuscript, read, and agreed to the published version of the manuscript.

## Conflict of Interest

The authors declare that the research was conducted in the absence of any commercial or financial relationships that could be construed as a potential conflict of interest.
